# The Impact and Applications of Social Media Platforms for Public Health Responses Before and During the COVID-19 Pandemic: Systematic Literature Review

**DOI:** 10.2196/33680

**Published:** 2022-04-11

**Authors:** Dinesh Visva Gunasekeran, Alton Chew, Eeshwar K Chandrasekar, Priyanka Rajendram, Vasundhara Kandarpa, Mallika Rajendram, Audrey Chia, Helen Smith, Choon Kit Leong

**Affiliations:** 1 National University of Singapore Singapore Singapore; 2 University College London London United Kingdom; 3 University of Rochester Medical Center New York, NY United States; 4 Ministry of Health Holdings Singapore Singapore; 5 Lee Kong Chian School of Medicine Nanyang Technological University Singapore Singapore Singapore; 6 Mission Medical Clinic Singapore Singapore

**Keywords:** digital health, social media, big data, population health, blockchain, COVID-19, review, benefit, challenge, public health

## Abstract

**Background:**

Social media platforms have numerous potential benefits and drawbacks on public health, which have been described in the literature. The COVID-19 pandemic has exposed our limited knowledge regarding the potential health impact of these platforms, which have been detrimental to public health responses in many regions.

**Objective:**

This review aims to highlight a brief history of social media in health care and report its potential negative and positive public health impacts, which have been characterized in the literature.

**Methods:**

We searched electronic bibliographic databases including PubMed, including Medline and Institute of Electrical and Electronics Engineers Xplore, from December 10, 2015, to December 10, 2020. We screened the title and abstracts and selected relevant reports for review of full text and reference lists. These were analyzed thematically and consolidated into applications of social media platforms for public health.

**Results:**

The positive and negative impact of social media platforms on public health are catalogued on the basis of recent research in this report. These findings are discussed in the context of improving future public health responses and incorporating other emerging digital technology domains such as artificial intelligence. However, there is a need for more research with pragmatic methodology that evaluates the impact of specific digital interventions to inform future health policy.

**Conclusions:**

Recent research has highlighted the potential negative impact of social media platforms on population health, as well as potentially useful applications for public health communication, monitoring, and predictions. More research is needed to objectively investigate measures to mitigate against its negative impact while harnessing effective applications for the benefit of public health.

## Introduction

Humans are an inherently social species, and the evolutionary and health benefits of this trait are well documented [1]. This predilection to form and live in groups is deeply rooted in human psychology. It follows that the fourth industrial revolution of digitization has brought with it social platforms as a technological embodiment of human interconnectedness and communication. Social media platforms bring content sharing and entertainment to the masses. They superficially bridge time and space to enable friendship, intimacy, and a sense of connection, consuming the time and attention of most individuals across all ages on a daily basis [2,3]. However, the COVID-19 pandemic has revealed the downside of this “online closeness,” as with the greater ease of infectious disease transmission from physical closeness [4,5].

Social media platforms have drawn criticism for propagating misinformation and crowding out of public health communication [6,7]. As the pandemic rages on, it has exposed our limited knowledge regarding the potential health impact of these platforms, which have been a medium to propagate false information and widespread population anxiety [8,9]. It is timely, therefore, to investigate the benefits and drawbacks of social media on population health [10]. In this review, we aim to highlight a brief history of social media in health care, its negative public health impact that has marred outbreak responses, and its potential positive impact.

## Methods

We searched electronic bibliographic databases, including PubMed, including Medline and Institute of Electrical and Electronics Engineers Xplore, from December 10, 2015, to December 10, 2020, with the following search terms: “((Social media[Title/Abstract]) OR (Social network[Title/Abstract]) OR (TikTok[Title/Abstract]) OR (Facebook[Title/Abstract]) OR (Instagram[Title/Abstract]) OR (Twitter[Title/Abstract]) OR (Baidu[Title/Abstract]) OR (Weibo[Title/Abstract])) AND ((Public health[Title/Abstract]) OR (Infectious Disease[Title/Abstract]) OR (Outbreak[Title/Abstract]) OR (Pandemic[Title/Abstract]) OR (COVID[Title/Abstract])) AND ((Intervention[Title/Abstract]) OR (Content analysis[Title/Abstract]) OR (Trial [Title/Abstract]) OR (Application[Title/Abstract]) OR (Health Promotion [Title/Abstract])) AND (English[Language]).”

A total of 678 reports were identified. We screened the title and abstract of these reports to identify relevant English-language manuscripts for this review. The full text of selected manuscripts and their reference lists were analyzed thematically on the basis of the thematic paradigm of social media apps for public health communication, monitoring, and predictions. This analysis was conducted by a multidisciplinary panel of clinicians, researchers, public health specialists, and professors from business and medical schools to provide a holistic assessment of the published literature. The findings of this panel based on the reviewed studies are described using a narrative review approach in accordance with specific issues described in the literature, which have a positive or negative impact on population health, in order to inform future public health responses.

## Results

### Social Media: A Brief History Before the COVID-19 Pandemic

**Figure 1:**
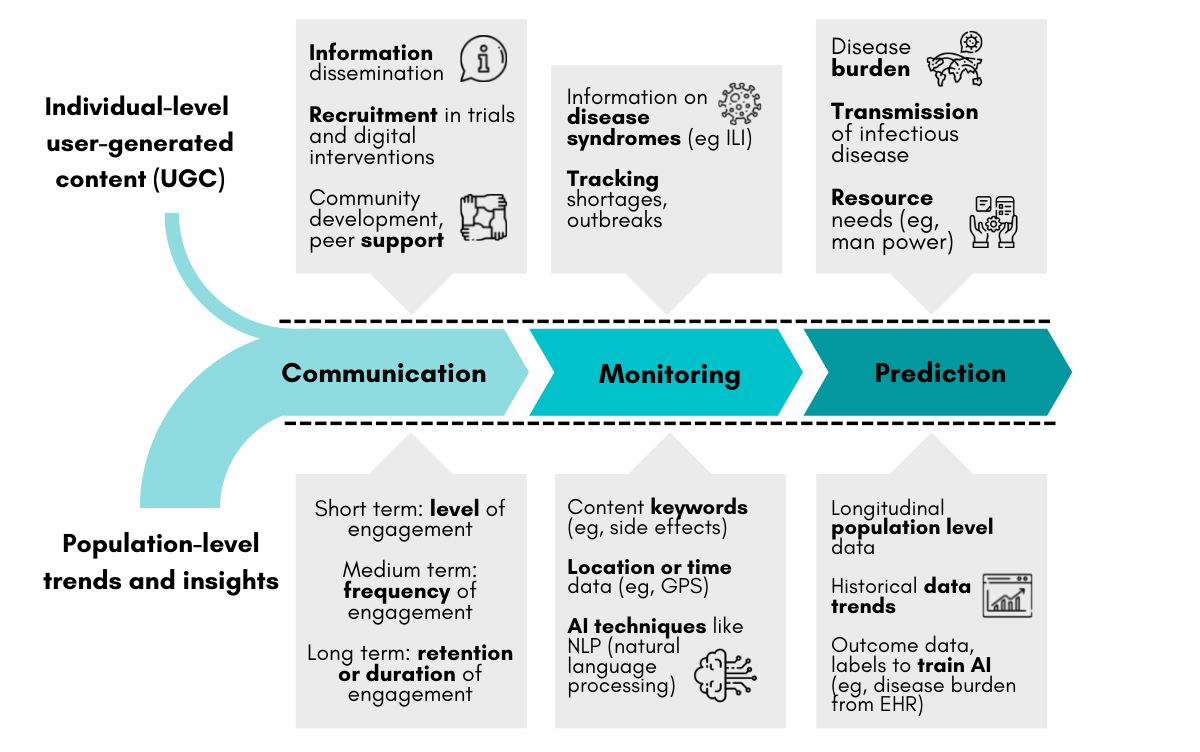
Evaluating the impact and applications of social media in public health. AI: artificial intelligence, EHR: electronic health record, ILI: influenza-like illness.

Prior to the COVID-19 pandemic, the possibilities of scalable public health promotion through leveraging the network effects of social media had garnered praise from the academic community. The effectiveness of these platforms for dissemination of information, conduct of digital interventions, or individual campaigns can be evaluated at three levels of chronology. These include the short-term using level of engagement (frequency or duration a platform is accessed each day, number of reactions or shares to content, etc), medium-term with frequency of engagement (daily or monthly active users, etc), and long-term based on retention or duration of engagement (adherence to or compliance with digital interventions) [11,12]. The ubiquity of social media platforms enables many public health applications including the communication of public health messages, real-time monitoring of population health, and potential predictions such as infectious disease outbreaks [13]. Descriptions of these applications in existing literature are summarized thematically and depicted in Figure 1.

#### Communication: Digital Public Health Promotion

The exponential potential of social media platforms for information dissemination has been strategically used for positive impact in the past [3]. They can be applied to reinvigorate public health promotion efforts and raise awareness about diseases, as exemplified by the “ALS Ice Bucket Challenge” in 2014 [14]. Other such health promotion campaigns include smoking cessation campaigns such as Tweet2Quit [12], and the #smearforsmear campaign to raise awareness about cervical cancer screening [15]. These initiatives capitalize on the network effects of social media to amplify the impact of web-based public health interventions. This is achieved by leveraging visibility (through search or content), peer-to-peer advocacy (“word of mouth”), or contextual paid advertising, the fundamental pillars of marketing digital initiatives [16,17].

The Tweet2Quit initiative attracted considerable attention to public health promotion using social media following a randomized controlled trial of a digital intervention using Twitter to help smokers abstain from their habit. It recruited users into twitter groups of 17-20 participants and encouraged smoking cessation by seeding conversation topics for users using automated messages to each group. These messages were aligned with clinical practice smoking cessation guidelines. The messages served as a conversation starter for users to provide encouragement for others, forging camaraderie as they embarked on their arduous smoking cessation journeys. The digital intervention was found to be more effective than Nicotine patches and a quit smoking website in this study [18].

#### Monitoring: Precision Public Health

The epidemiological value of social media applications includes surveillance of information, disease syndromes, and events (outbreak tracing, needs or shortages during disasters) [19]. The benefit of social media is that it provides real-time big data rapidly to epidemiologists from millions of users worldwide. The utility of epidemiological monitoring using social media during public health emergencies was well illustrated in the H7N9 avian influenza A virus outbreak using user-generated content (UGC) in the Sina microblog and the daily Baidu Activity Index [20]. This facilitated network monitoring for rapid information collection, allowing officials to disseminate public health communication in a relevant and timely manner when information-seeking behavior was at its highest. These methods were reproduced following subsequent outbreaks, highlighting potential rapid surveillance of population reactions to outbreaks and informing public health responses [21,22].

During the initial onset of an outbreak, uncertainty promotes fear among members of the public, who become desperate for more information. During the H1N1 outbreak: internet attention peaked in the first 3 days before dwindling as information saturation set in [23]. Public attention was also positively correlated with the case fatality rate and geographical advancement of the outbreak. This suggests that public health communication should use such critical features and time points in an outbreak to draw attention to accurate information. To achieve this, social media seems to present a potential tool for governments to (1) rapidly assess public reaction to an outbreak, (2) identify critical time points and topics that need to be addressed, and (3) rapidly disseminate vital public health communication during outbreaks.

During the 2009 H1N1 outbreak, real-time monitoring using Twitter was clearly demonstrated [23]. Tweets containing relevant keywords such as “flu,” “swine,” and “Tamiflu” among others were extracted along with geolocations and time stamps. These were largely found to be posted by users in Twitter “live” (ie, real-time) and often contained information about the users’ condition or symptoms. The researchers then applied a machine learning method to create a real-time model for the estimation of disease activity from the data, and demonstrated positive correlation with the national and regional prevalence of influenza-like illness reported by the US Centre for Disease Control and Prevention’s (CDC’s). They proposed other potential applications of social media using similar techniques, including surveillance for treatment side effects and shortages in medical supplies.

#### Predictions: Public Health Forecasting and Planning

Infodemiology (ie, information epidemiology) entails methods which analyze trends in web-based health data for applications, such as policy making [24,25]. On the other hand, infoveillance (ie, information surveillance) is the detection of events using web-based data, which can be faster than traditional surveillance methods [26,27]. Earlier studies have successfully illustrated the use of social media microblogs and geographical locations to track infectious disease outbreaks in many countries [28]. The authors searched Twitter for keywords such as “headache,” “fever,” and “runny nose” among others, mapping the locations of these tweets against the results of CDC’s surveillance system FluView. They then modeled the potential spread of influenza on the basis of airline traffic and demonstrated predictions of influenza outbreaks a week in advance. The technical demonstration of these capabilities was a prelude to their future application during the current pandemic, which are discussed in a later section on social media and the COVID-19 pandemic.

### Social Media and Infodemics

Although social media has the potential for positive public health utility, it can also amplify poor quality content [3]. Public fear and anxiety are known to be heightened by sensational reporting in the media during outbreaks, a phenomenon heightened by the ease of sharing on social media. These trends were described during previous outbreaks, such as the prominence of risk-elevating messages in American media during the Ebola outbreak [29]. During the COVID-19 pandemic, cross-sectional surveys in Russia, Bangladesh, and Iraq found elevated baseline levels of anxiety in individuals with higher levels of consumption of COVID-19–related news [8,30,31].

Similar associations between media consumption and mental health disorders have been reported during the COVID-19 pandemic and were worsened by poor quality of information dissemination among quarantined undergraduates in France [32]. The sharing of poor-quality information during outbreaks was also highlighted in an earlier infodemiological analysis of public reactions to the Zika epidemic between 2015 and 2016. Reliable sources, including the World Health Organization, had accounted for less than 0.1% of all highest-ranking content by dissemination, while over a quarter originated from social media like Facebook and Twitter. A similar study conducted during the COVID-19 pandemic suggested improved prominence of reliable sources [33], which may be driven by technology and public health partnerships for education campaigns [3,34].

### The Impact of Social Media During the COVID-19 Pandemic

Despite the negative impact of social media in propagating “infodemics,” it also provides a reservoir of UGC as individuals share a range of topics from emotions to symptoms [35]. The COVID-19 pandemic has shed light on various public health applications of social media that were developed and piloted post hoc using retrospective data such as trends in UGC following earlier outbreaks [36]. However, the potential real-world impact of several applications of social media platforms as digital health interventions have been hindered by a lack of stakeholder engagement and barriers to adoption [37]. The following section summarizes descriptions of social media apps for public health communication, monitoring, and predictions during the COVID-19 pandemic.

#### Communication During the COVID-19 Pandemic

The volumes of fear-driven information sharing at the beginning of the pandemic overwhelmed individuals and the capacity of regulators in many regions [7]. Some distributors capitalized on public anxiety, using fear mongering and predatory sales tactics for fraudulent medical products, a situation worsened by high-profile figures touting baseless claims [6,38]. Moreover, professionals were bombarded with rapidly evolving advisories as public health organizations and academia scrambled to process the flood of scientific reports of variable quality [39]. These challenges have presented an unprecedented need for scalable tools that detect trends in information sharing, develop targeted public health communications, and facilitate their dissemination—both to members of the public as well as frontline health care workers [5].

Fortunately, new methods using topical modeling and engagement metrics in social media were available to allay the concerns of the public and provide updated information to health care professionals. These leveraged application programming interfaces (APIs) of platforms such as Twitter or Weibo to identify trends in content sharing to inform public health communications [34,40]. These methods used such APIs to filter UGC on the basis of predetermined hashtags as well as identify temporal or geographical trends in information sharing, topical modeling, and engagement using the natural language processing branch of artificial intelligence (AI) [33]. Reports have also described pairing these techniques with sentiment analysis using Python textblob library or Valence Aware Dictionary and sEntiment Reasoner to provide a barometer of public sentiment [41,42].

Finally, social media has also been applied as a tool for grassroots health promotion initiatives [3,43]. For example, many US physicians actively used Twitter for health promotion during the COVID-19 pandemic [44], and in Singapore, the Government applied various social media platforms for health promotion initiatives [5]. In Italy, these platforms were even used by health care professionals to share practice updates and scientific information among one another [45]. Despite these benefits, the relative lack of legitimate voices has been thought to enable the propagation of misinformation in social media, such as the purported association between the COVID-19 pandemic and 5G networks in the United Kingdom [46]. Nonetheless, sometimes misinformation has been aggravated by academics or health care providers stepping outside their areas of expertise in well-meaning attempts to help educate the public [47].

#### Monitoring Applications During the COVID-19 Pandemic

Comprehensive surveillance is vital during infectious disease outbreaks to monitor compliance and effectiveness of measures such as social distancing [48]. This allows the extensiveness of these measures to be tailored, to balance competing individual freedoms, health, and economic priorities for public benefit [48]. During the COVID-19 pandemic, methods leveraging social media, which were validated in previous outbreaks, were applied prospectively to inform advisories for both health care practitioners and public health administrators. Social reactions on Instagram and Twitter can be used as proxies for outbreak monitoring and assessment of public health measures for outcomes such as reduction in the basic reproductive number of COVID-19 with social distancing, as demonstrated in the United States [49].

This underscores the importance of investigating the relationship between web-based and offline behavior for translatable population health benefits. Digital data from social media platforms has also been used to detect predatory sellers, counterfeit health products, and unapproved products with questionable claims [50]. These emerging applications can provide governments and health authorities effective tools for real-time monitoring of public health measures, targeted law enforcement activities, and developing protective measures for public safety.

#### Prediction Techniques Applied During the COVID-19 Pandemic

The AI and regression techniques applied for the abovementioned real-time monitoring applications were based on cross-sectional data that became available during the pandemic. However, increases in computational power and availability of large, longitudinal data sets have paved the way for applications of big data from social media for future outbreak forecasting among other predictions. Applications that predict the potential number of cases during the COVID-19 outbreak used social media search indexes (SMSI) for keywords such as “dry cough,” “fever,” “coronavirus,” and “pneumonia” on platforms such as Baidu, where a significant correlation between new COVID-19 cases and SMSI findings have been reported [51].

Researchers have even developed and demonstrated such capabilities during the COVID-19 pandemic to accurately predict the burden of incident cases 2 weeks ahead of official sources [52]. This was achieved by applying the machine learning (ML) branch of AI to the social media posts of over 250 million users of the Weibo social media platform, based on self-reported symptoms and illness in UGC. The scale of big data and predictive value of novel such approaches represent a paradigm shift for public health capabilities, enabling anticipatory strategies and agile infection control responses driven by real-world data during an evolving threat [9].

However, it is worth noting that the CDC’s prediction initiative COVID-19 forecast hub has indicated that methods using social media big data have underperformed traditional methods such as the Susceptible-Exposed-Infectious Removed (SEIR) model when applied for forecasting. Ultimately, further research is necessary to fine-tune these novel techniques, and researchers may find that applying social media apps together with existing traditional modeling paradigms such as the SEIR may yield superior results. Limitations of existing modeling approaches include their primary focus on human-human transmission, along with difficulties modelling environmental transmission from fomites as well as variations in transmissibility. The latter is particularly important in a new public health emergency with growing awareness over time and public health communication such as that to encourage the adoption of hygiene measures. Public health organizations may also consider funding this research for capacity building to evaluate how these tools can be applied to enhance resource allocation during future health crises.

## Discussion

### Principal Findings

The COVID-19 pandemic has exposed the public health risks of unchecked health information–sharing on social media. It has also highlighted the pivotal role of human behavior in epidemic risk, prevention, and control [53]. This review has highlighted the potential negative impact of social media platforms on population health, as well as their useful public health applications for communication, monitoring, and predictions. Strategic planning for outbreaks should specifically explore leveraging the benefits of social media as potential tools for public health responses, as well as specific measures to mitigate against its potential drawbacks [9]. This includes planned behavioral and social communication to mitigate the infodemic, along with monitoring and predictive applications identified in this review. To be most effective, this needs to be developed using a participatory approach involving members of target populations [53,54].

The literature regarding social media apps for public health communication before the COVID-19 pandemic highlighted that digital behavioral modifications can be less time-consuming and less costlier than traditional approaches implemented using offline channels, such as patient support groups [55]. Through the rapidity and ease of recruitment facilitated by social media, these studies have shed light on the potential for social media to be applied in a scalable manner for behavioral modifications through peer support and networks. Other benefits include ease of monitoring and withdrawing these trials, as well as low inherent risks to participants given the use of platforms that are already widely used. Nonetheless, as with any patient-directed digital intervention, risk mitigation measures such as methodology and ethics review are critical to ensure participants understand the intervention, potential benefits, and its risks [56].

Although similar applications of social media for communication were effectively applied to amplify public health messages during the COVID-19 pandemic, they were also used by some to perpetuate the spread of misinformation, thus marring its positive impact [47]. Therefore, researchers are now calling for novel approaches such as provider-moderated online health communities (OHCs), to leverage the utility of social networks as scalable channels for the dissemination of information, with added controls such as expert peer-verification to amplify benefits over its risks [57]. OHCs have been developed by social entrepreneurs to connect stakeholders such as health experts, providers, caregivers, and patients on a common platform. Besides OHCs, social entrepreneurs have also devised frugal solutions to successfully address a range of public health problems such as last-mile health, sanitation, and capacity building of health care workers [58]. Other relevant applications for health include disease-focused virtual communities, such as ParkinsonsNet, topical forums within Reddit, and the Psoriasis MSN or Google groups [59,60]. General health forums such as WebMD communities have also been previously described [61]. New OHCs have since been created for clinical practice updates, ranging from individual clinical discussions to entire virtual conferences. These include the inaugural virtual Primary Care Grand Conference (AGC) launched on the internet in Singapore in 2020 by an OHC, with participation of stakeholders from various sectors—clinical, allied health, political, and social sectors—to provide comprehensive updates on trends in disease presentation and administration [62].

New trends in personal content creation are constantly emerging, such as video logging (“vlogging”) using platforms such as TikTok or modules of established platforms such as “Stories” in Instagram [63]. These present new challenges for regulation. Content moderation is especially challenging given the size of video content and configurations with automated purging after a brief interval during which many impressions can be formed. This can happen rapidly at scale, creating a narrow window for enforcement. Users of these platforms are disproportionately represented by youth, and their demand for health-related content producers exceed their supply, exposing vulnerable users to content from sources of uncertain reliability [63].

Nonetheless, various reports of these public health responses to the COVID-19 pandemic, which applied social media for positive impact signal a future in which these platforms can be used to address new public health threats. Social media data can be combined with other sources of publicly available and digital behavioral data to improve the accuracy of existing approaches for various public health applications. These include analysis of UGC in open social media platforms such as Twitter, as well as internet search trends in search engines using ML. This has been demonstrated for applications such as monitoring for influenza surveillance [64]. Data from social media applications for public health monitoring in this manner can be used for operational planning. This is particularly useful when triangulated with other sources of data pertaining to web-based behavior, such as search trends, which have been used to predict future requirements for telehealth capacity [65]. These were also introduced during the COVID-19 pandemic for the monitoring of social distancing measures [49] and predictions of outbreaks [66].

However, the effectiveness of applying these tools at a population level has yet to be formally evaluated [36]. Moreover, given the heterogeneity between these social media platforms and within them as they evolve over time, another future area for further research would be to evaluate the public health implications of specific modules or social media functions. These remain key priorities for future research to improve our understanding of this new digital domain within public health. New variables of interest in outbreaks, such as attention saturation with temporal and topical variation, were already described in one such multinational study corroborating multimodal content from Reddit, Wikipedia, and news media with epidemic progression [67]. Furthermore, data from electronic medical records (EMRs) have been previously applied through global disease registries to improve our understanding of infectious diseases [68,69]. The incorporation of data from EMRs to triangulate with publicly available data can also improve the validity of these tools [70]. However, each additional source of data carries privacy concerns that must be addressed [71].

Future research is needed to develop scalable methods to mitigate against the risks of “online closeness.” Fortunately, solutions such as provider-moderated OHCs have emerged as potential tools to counter web-based medical misinformation, with applications described in fields such as psychiatry [57] and anesthesia for chronic pain management during the COVID-19 pandemic [72]. Moreover, promising solutions to automate the processing of big data using the deep learning branch of AI, such as long short-term memory or gated recurrent unit neural networks are emerging [60,73]. Finally, progress in cryptography has resulted in the successful incorporation of blockchain in digital platforms [74]. These are distributed databases with security configurations accommodating smart contracts, and programmable permissions for data access [75,76]. These configurations help address privacy concerns with data accession for the applications of social media big data by only revealing aggregated trends for public health planning while concealing individual identity through cryptography, or enabling the incorporation of individual consent for data accession transparently using smart contracts. Relevant applications of blockchain in health care during the COVID-19 pandemic include real-time tracking of drug delivery following telemedicine services [74]. The field of blockchain is still evolving with new potential applications such as social money solutions that provide financial incentives for content creation [77], and could be applied to reward creators of reliable content to combat web-based medical misinformation. Other potential health care applications include programmable patient consent or compute-to-data solutions for privacy-preserving data applications [78,79], data storage in medical devices [80], and queries from pharmacogenomics databases [81].

The incorporation of these various technologies with social media platforms may eventually contribute to a “learning” digital public health system in future, that can scale up and improve existing methods for targeted communication, monitoring, and predictions [60]. However, improved study design and more empirical investigations of specific digital interventions using social media platforms are needed to develop and validate targeted strategies for key responses. For instance, recent reports described the use of programmed reminders to prompt individuals to consider accuracy of UGC [82]. Others forged partnerships between formal news media and social media influencers for targeted health promotion campaigns [43].

Finally, strategies for implementing these tools in health care macrosystems will also need to be developed. Examples of these include implementation of these tools using the lighthouse and safety net operational models for remote monitoring solutions [37]. Ultimately, more research on the link between health and human behavior is urgently required.

### Conclusions

The pandemic has had a massive human toll and economic impact [83]. However, even with the availability of vaccines, new challenges remain including problems of logistics, distribution to low-income nations, and antivaccine activism [6,84]. Fortunately, social media platforms have emerged as new digital tools for public health professionals and providers. This review has highlighted existing and developing applications of social media for public health communication, monitoring, and predictions. These tools were sharpened by our experience with the COVID-19 pandemic and will likely have increasing prominence in responses to future public health threats. However, we also identified a need for greater pragmatic research for these applications of social media in order to better inform public health responses.

## Abbreviations

AI: artificial intelligence
API: application programming interface
CDC: US Centers for Disease Control and Prevention
EMR: electronic medical record
LKCMedicine: Lee Kong Chian School of Medicine
ML: machine learning
NUS: National University of Singapore
OHC: online health community
SIER: Susceptible-Exposed-Infectious Removed
SMSI: social media search indexes
UGC: user-generated content
